# Automatic structuring of radiology reports with on-premise open-source large language models

**DOI:** 10.1007/s00330-024-11074-y

**Published:** 2024-10-10

**Authors:** Piotr Woźnicki, Caroline Laqua, Ina Fiku, Amar Hekalo, Daniel Truhn, Sandy Engelhardt, Jakob Kather, Sebastian Foersch, Tugba Akinci D’Antonoli, Daniel Pinto dos Santos, Bettina Baeßler, Fabian Christopher Laqua

**Affiliations:** 1 Department of Diagnostic and Interventional Radiology, University Hospital Würzburg, Würzburg, Germany; 2 Department of Diagnostic and Interventional Radiology, University Hospital Aachen, Aachen, Germany; 3 Department of Internal Medicine III, Heidelberg University Hospital, Heidelberg, Germany; 4 DZHK (German Centre for Cardiovascular Research), Partner Site Heidelberg/Mannheim, Heidelberg, Germany; 5 Department of Internal Medicine I, University Hospital Carl Gustav Carus, Technical University Dresden, Dresden, Germany; 6 Else Kroener Fresenius Center for Digital Health, Medical Faculty Carl Gustav Carus, TUD Dresden University of Technology, Dresden, Germany; 7 Medical Oncology, National Center for Tumor Diseases (NCT), University Hospital Heidelberg, Heidelberg, Germany; 8 Institute of Pathology, University Medical Center Mainz, Mainz, Germany; 9 Institute of Radiology and Nuclear Medicine, Cantonal Hospital Baselland, Liestal, Switzerland; 10 Department of Diagnostic and Interventional Radiology, University of Cologne, Cologne, Germany; 11 Department of Radiology, University Hospital of Frankfurt, Frankfurt, Germany

**Keywords:** Structured reporting, Large language models, Chest radiography

## Abstract

**Objectives:**

Structured reporting enhances comparability, readability, and content detail. Large language models (LLMs) could convert free text into structured data without disrupting radiologists’ reporting workflow. This study evaluated an on-premise, privacy-preserving LLM for automatically structuring free-text radiology reports.

**Materials and methods:**

We developed an approach to controlling the LLM output, ensuring the validity and completeness of structured reports produced by a locally hosted Llama-2-70B-chat model. A dataset with de-identified narrative chest radiograph (CXR) reports was compiled retrospectively. It included 202 English reports from a publicly available MIMIC-CXR dataset and 197 German reports from our university hospital. Senior radiologist prepared a detailed, fully structured reporting template with 48 question-answer pairs. All reports were independently structured by the LLM and two human readers. Bayesian inference (Markov chain Monte Carlo sampling) was used to estimate the distributions of Matthews correlation coefficient (MCC), with [−0.05, 0.05] as the region of practical equivalence (ROPE).

**Results:**

The LLM generated valid structured reports in all cases, achieving an average MCC of 0.75 (94% HDI: 0.70–0.80) and F1 score of 0.70 (0.70–0.80) for English, and 0.66 (0.62–0.70) and 0.68 (0.64–0.72) for German reports, respectively. The MCC differences between LLM and humans were within ROPE for both languages: 0.01 (−0.05 to 0.07), 0.01 (−0.05 to 0.07) for English, and −0.01 (−0.07 to 0.05), 0.00 (−0.06 to 0.06) for German, indicating approximately comparable performance.

**Conclusion:**

Locally hosted, open-source LLMs can automatically structure free-text radiology reports with approximately human accuracy. However, the understanding of semantics varied across languages and imaging findings.

**Key Points:**

***Question*** *Why has structured reporting not been widely adopted in radiology despite clear benefits and how can we improve this?*

***Findings*** *A locally hosted large language model successfully structured narrative reports, showing variation between languages and findings.*

***Critical relevance*** *Structured reporting provides many benefits, but its integration into the clinical routine is limited. Automating the extraction of structured information from radiology reports enables the capture of structured data while allowing the radiologist to maintain their reporting workflow.*

## Introduction

Structured reporting is broadly defined as using software to import and arrange medical content in a radiological report [1]. It has established itself in radiology as an effective tool for standardizing medical terminology, improving communication with clinicians, enabling integration with artificial intelligence (AI) systems and clinical parameters, and optimizing radiologists’ workflow [2]. Structured data support research by simplifying processes of extracting well-organized labels for AI models, streamlining data retrieval, regulatory compliance, and automated analytics [3].

According to Nobel et al [1], free-text reports can be structured at two levels: the first involves a structured layout with a standardized order of findings and fixed headings. The second level includes structured content, featuring elements like dropdown menus, gaps to fill, pick lists, and flowchart guidance. Fully structured reports, where findings and their attributes are selected from predefined options—representing the second level of structuring—might offer less flexibility but are significantly more straightforward to mine and use in research and analytics use cases [4]. Despite the interest from radiologists, structured reporting only gained limited adoption in clinics due to its drawbacks. These include resistance to changing existing efficient workflows, inflexibility of templates, and insufficient customization options [5]. Furthermore, structured reporting might be more time-consuming than traditional reporting, requires specialized training, and involves a learning curve [2].

Large language models (LLMs) present a promising approach to overcome those challenges and increase the adoption of structured reporting. LLMs are AI systems trained on large amounts of texts derived from articles, books, and other digital content. Recently released Generative Pre-trained Transformer (GPT) models, including ChatGPT and GPT-4, have revolutionized text processing and exhibited near-human-level performance in various cognitive tasks, including those in the medical domain [6, 7]. They have been successfully applied to multiple tasks in radiology, such as board question answering, protocol selection, patient education, differential diagnosis, document pre-authorization, and, in particular, structuring radiology reports [8]. However, their use requires sending data to the cloud, which raises privacy concerns in the healthcare domain. In July 2023, Meta AI published Llama-2 [9], a collection of powerful LLMs, under a license permissive for research and commercial applications. The research community enthusiastically adopted these models, creating numerous open-access tools, which have amplified their capabilities, making them more effective and offering more fine-grained control. Contrary to GPT-4, these models could be deployed locally in a privacy-preserving fashion, their output could be controlled more easily, and the context could be cached more effectively [10].

Post-hoc extraction of structured information with LLMs might allow radiologists to maintain their traditional reporting workflow while capturing structured data. Recent research in this domain mainly focused on first-level structuring [11, 12], without extracting fully structured content. Studies often used commercial models like GPT-4 [11, 12] that require sending sensitive patient data to a cloud server for processing, raising concerns regarding patient privacy and data protection [7].

In this study, we developed a novel approach for automatically structuring free-text radiology reports using a locally hosted, open-source LLM. Our approach preserves privacy and guarantees the validity of the structured reports. We evaluated it on two datasets of chest radiography reports in English and German, including publicly available and internal reports.

## Materials and methods

### Study design and data preparation

This study was conducted in compliance with the Declaration of Helsinki, and the need for informed written consent was waived by the Institutional Review Board (IRB approval number: 20221004-02). Chest radiography free-text reports were retrospectively acquired from two sources: (1) the publicly available MIMIC-CXR dataset [13] (https://physionet.org/content/mimic-cxr/2.0.0, accessed 12.09.2023) and (2) our institution, further referred to as the University Hospital (UH). MIMIC-CXR includes de-identified English reports for chest radiographs performed at the Beth Israel Deaconess Medical Center between 2011 and 2016. Meanwhile, reports from UH, written in German by various board-certified radiologists, span from January 2015 to August 2022, and were extracted directly from the radiology information system. We randomly sampled 300 reports from each data source. Each report was de-identified manually if necessary, and only findings-related sections were used. Reports without any pathological findings were excluded to increase the proportion of positive findings relative to negative ones. Finally, 202 reports from MIMIC-CXR and 197 from UH were included in the analysis.

**Fig. 1:**
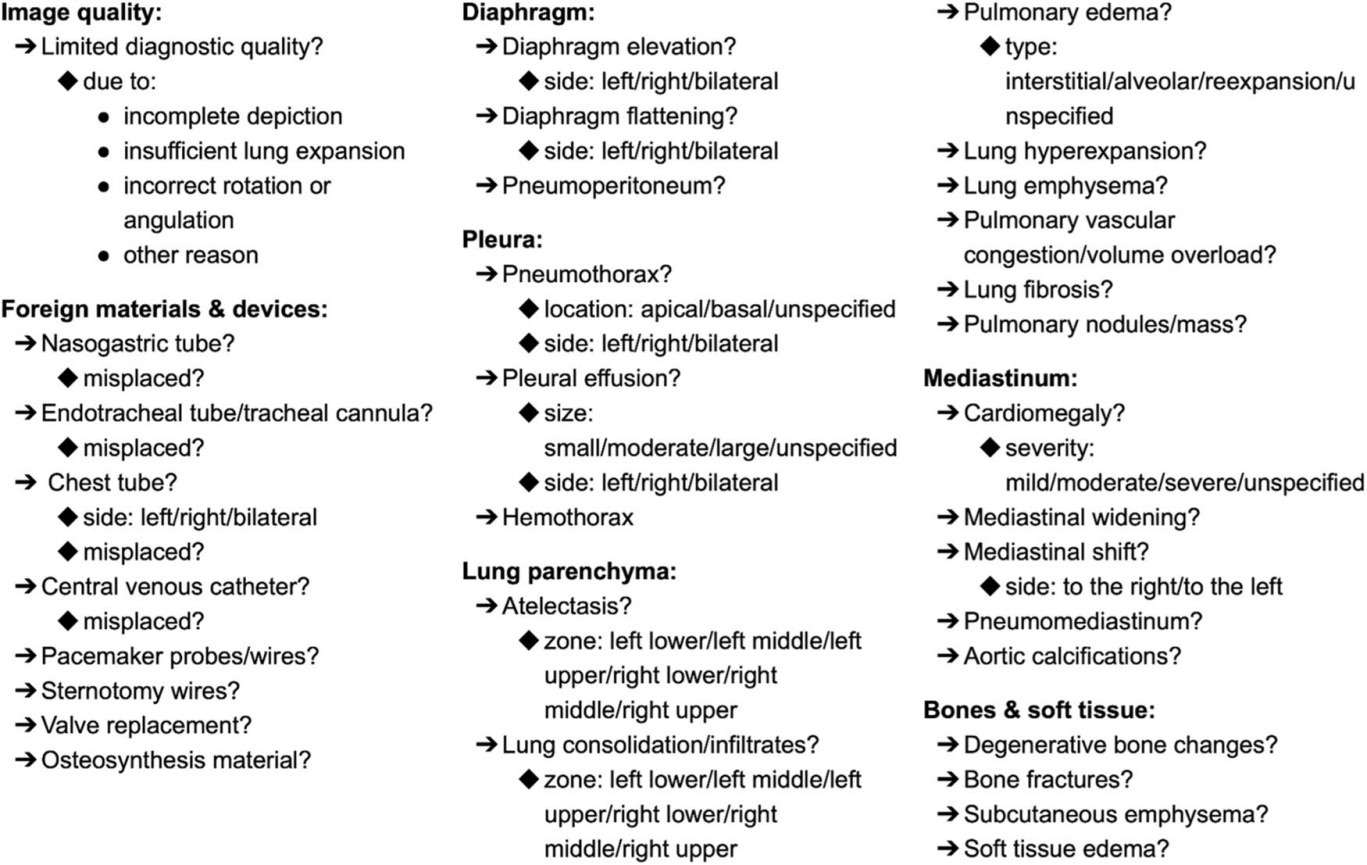
Reporting template. The template consists of 48 question-answer pairs and includes questions with a binary answer (possible answers: finding present or absent, hidden for clarity), marked with question marks, and questions with specified answer options, marked with a colon (possible answers provided after the colon). The template includes nested questions, answered only if the parent finding is present

A senior radiologist (B.B.) with 12 years of experience in cardiothoracic imaging and extensive experience in structured reporting devised a comprehensive, nested template with 48 structured elements grouped into seven sections (Fig. 1). The template was structured as a list of question-answer pairs, with 34 closed yes/no questions indicating the presence or absence of a finding, eleven questions with multiple answer options and only one correct answer, and two questions with multiple correct answers. Therefore, the template corresponded to level two form structuring as defined by Nobel et al [1]. Thirty-two questions were asked for every report (top-level findings), the remaining sixteen only if the corresponding parent finding was present. For instance, question “Is pleural effusion present?” was answered for every report, and its presence or absence was a top-level finding, whereas question “On which side?” was asked only if pleural effusion was present.

### Report structuring

**Fig. 2:**
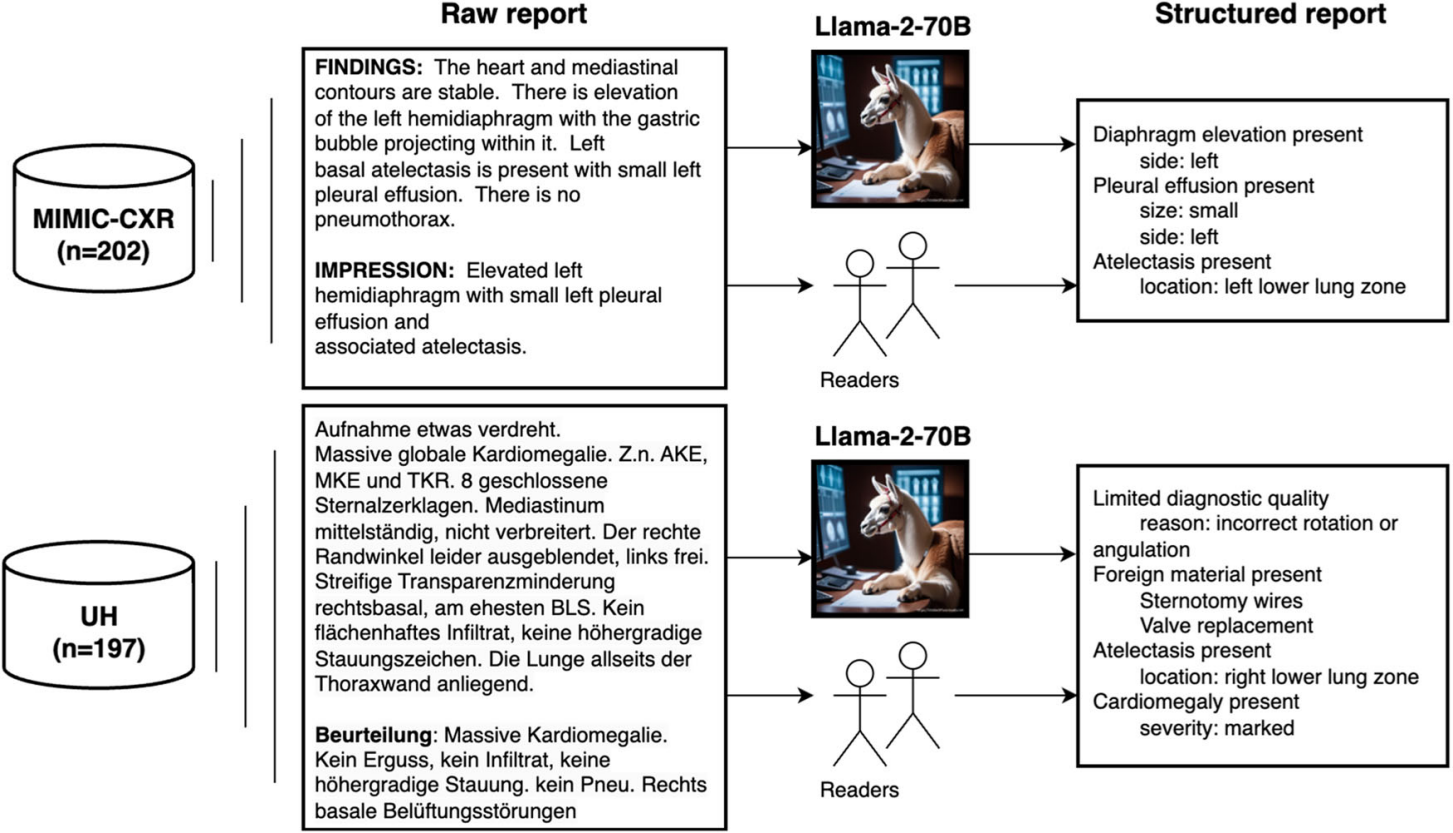
Study overview. Chest radiography reports from two sources were analyzed: MIMIC-CXR (English) and UH (German). The open-access Llama-2-70B model was used to extract structured elements from free-text radiology reports. The results of the automated structuring were compared with human readers. Llama-2-70B image was generated using GPT-4 through https://chat.openai.com/ on 11.11.2023. MIMIC-CXR, MIMIC chest X-ray cohort; UH, University Hospital cohort

Figure 2 presents an overview of the study workflow. The free-text reports were structured by both the LLM and human readers. Llama-2-70B-chat, the most powerful model from the Llama-2 family (further referred to as Llama-2-70B), was used for our experiments. We used the model from *HuggingFace* in the 8-bit quantization scheme. The model was run in a Docker container on a research server with two NVIDIA RTX 8000 GPUs, and an Intel Xeon Gold 5218R CPU with 128 GB RAM. It was provided with the following prompt:

*“You are Randy, a helpful radiology assistant, who helps extract relevant information from free-text radiology reports*.

*The following is a free-text report: <report>*

*Based on the report above fill out the following template, deciding if each finding is included in the report or not”*

The prompt was followed by the template to be filled out. The prompt and the template were formulated in English for both English and German reports. We used *Guidance*, a code library that enables fine control over the LLM output (*Lundberg S. (2024). Guidance (Version 0.0.64). GitHub*. https://github.com/guidance-ai/guidance). It allowed us to (1) prevent the LLM from giving “chatty” answers, (2) constrain the generation to a set of specified options, (3) answer downstream questions only if a particular option was selected, and (4) effectively cache context to speed up LLM generation. These properties guaranteed that extracted reports were always fully structured and valid.

In addition, two human readers (C.L., a 2nd-year cardiology resident, and I.F., a radiographer/MSc. Radiology with 8 years of experience) independently extracted the structured elements from all free-text reports, blinded to the reference standard annotations. The reference standard was established in consensus by a senior radiologist (B.B.) and a last-year radiology resident with 5 years of experience in cardiothoracic imaging (F.C.L.).

### Statistical analysis

We utilized Bayesian methods to evaluate the LLM classification performance against the human readers, incorporating uncertainty estimation. A hierarchical Bayesian network was constructed to model the probability distribution of the template elements and the associated contingency table [14, 15]. Multinomial distributions with Dirichlet priors were applied to model the distribution of each structured report element and the conditional probability distributions of correct classification, given the distribution in the reference standard. We defined uninformative hyperpriors. The No-U-Turn Sampler [16], a Markov chain Monte Carlo method, was applied to sample the posterior distributions of model parameters and derived secondary performance metrics (4 chains, 10,000 samples after 4000 burn-in samples). Model convergence was assessed using Rubin-Gelman statistics and visual trace plots. The micro-averaged Matthews correlation coefficient (MCC) was selected as the primary performance metric. In contrast to accuracy and F1, MCC takes into account all classes from the confusion matrix, providing measurable advantages in imbalanced datasets. It is also invariant to permutations of outcome labels [17]. F1 score, sensitivity, and specificity were used as secondary metrics. Inter-rater differences were assessed by analyzing the distribution of pairwise MCC differences.

The resulting metrics’ probability density distributions were characterized visually as ridge plots and quantitatively as the median, quartiles, and the 94% highest density interval (HDI). According to the framework of [15, 18], the region of practical equivalence for the MCC was defined as [−0.05, 0.05].

All statistical analyses were conducted in Python 3.11 using the *pymc* package with the *blackjax* backend. FCL is the statistical guarantor for this study. The source code for the statistical analyses and the experiments is available at https://github.com/baessler-lab/llm-tag (available after publication).

## Results

### Data characteristics

Cohort characteristics are presented in Table 1. The MIMIC-CXR cohort had a median report length of 61 words with a median of three pathological findings per report, whereas the UH cohort had a median of 67 words and four pathological findings per report. Forty-nine different clinicians wrote the radiology reports in the UH cohort. Frequencies of top-level template findings in study cohorts are presented in Table 2. The most common findings, with at least 20% prevalence in either cohort, were cardiomegaly (44% reports in both cohorts), pleural effusion (41% in MIMIC-CXR, 43% in UH), atelectasis (50% in MIMIC-CXR, 6% in UH), degenerative bone changes (7% in MIMIC-CXR, 41% in UH), aortic calcifications (8% in MIMIC-CXR, 33% in UH), diaphragm elevation (16% in MIMIC-CXR, 32% in UH), pulmonary venous congestion/volume overload (23% in MIMIC-CXR, 21% in UH), lung consolidations/infiltrates (23% in MIMIC-CXR, 13% in UH), and mediastinal widening (4% in MIMIC-CXR and 23% in UH).

**Table 1:** Cohort characteristics

	MIMIC-CXR (*n* = 202)	UH (*n* = 197)
Language	English	German
Exam time	2011–2016	2015–2022
Number of radiologists reporting	Not provided	49
Report length (words)	61 [21–182]	67 [18–171]
Number of pathological findings per report	3 [1–13]	4 [1–13]

Variables are reported as median and min-max range in brackets

*MIMIC-CXR* MIMIC chest X-ray cohort*, UH* University Hospital cohort

**Table 2:** Frequency of top-level findings in study cohorts

Finding	Frequency		Finding	Frequency	
	MIMIC-CXR	UH		MIMIC-CXR	UH
Limited diagnostic quality	0.03	0.05	Lung consolidation/infiltrates	0.23	0.13
Nasogastric tube	0.17	0.11	Pulmonary edema	0.19	0.04
Endotracheal tube/tracheal cannula	0.18	0.04	Lung hyperexpansion	0.06	0.04
Chest tube	0.08	0.11	Lung emphysema	0.04	0.14
Central venous catheter	0.19	0.18	Pulmonary vascular congestion/volume overload	0.23	0.21
Pacemaker wires	0.04	0.03	Lung fibrosis	0.01	0.03
Sternotomy wires	0.08	0.14	Pulmonary nodules/mass	0.04	0.04
Valve replacement	0.02	0.06	Cardiomegaly	0.44	0.44
Osteosynthesis material	0.04	0.03	Mediastinal widening	0.04	0.23
Diaphragm elevation	0.16	0.32	Mediastinal shift	0.02	0.07
Diaphragm flattening	0.03	0.03	Pneumomediastinum	0.01	0.02
Pneumoperitoneum	0.0	0.02	Aortic calcifications	0.08	0.33
Pneumothorax	0.08	0.13	Degenerative bone changes	0.07	0.41
Hemothorax	0.0	0.01	Bone fractures	0.02	0.06
Pleural effusion	0.41	0.43	Subcutaneous emphysema	0.02	0.05
Atelectasis	0.5	0.06	Soft tissue edema	0.03	0.02

*MIMIC-CXR* MIMIC chest X-ray cohort*, UH* University Hospital cohort

### Accuracy of report structuring

**Fig. 3:**
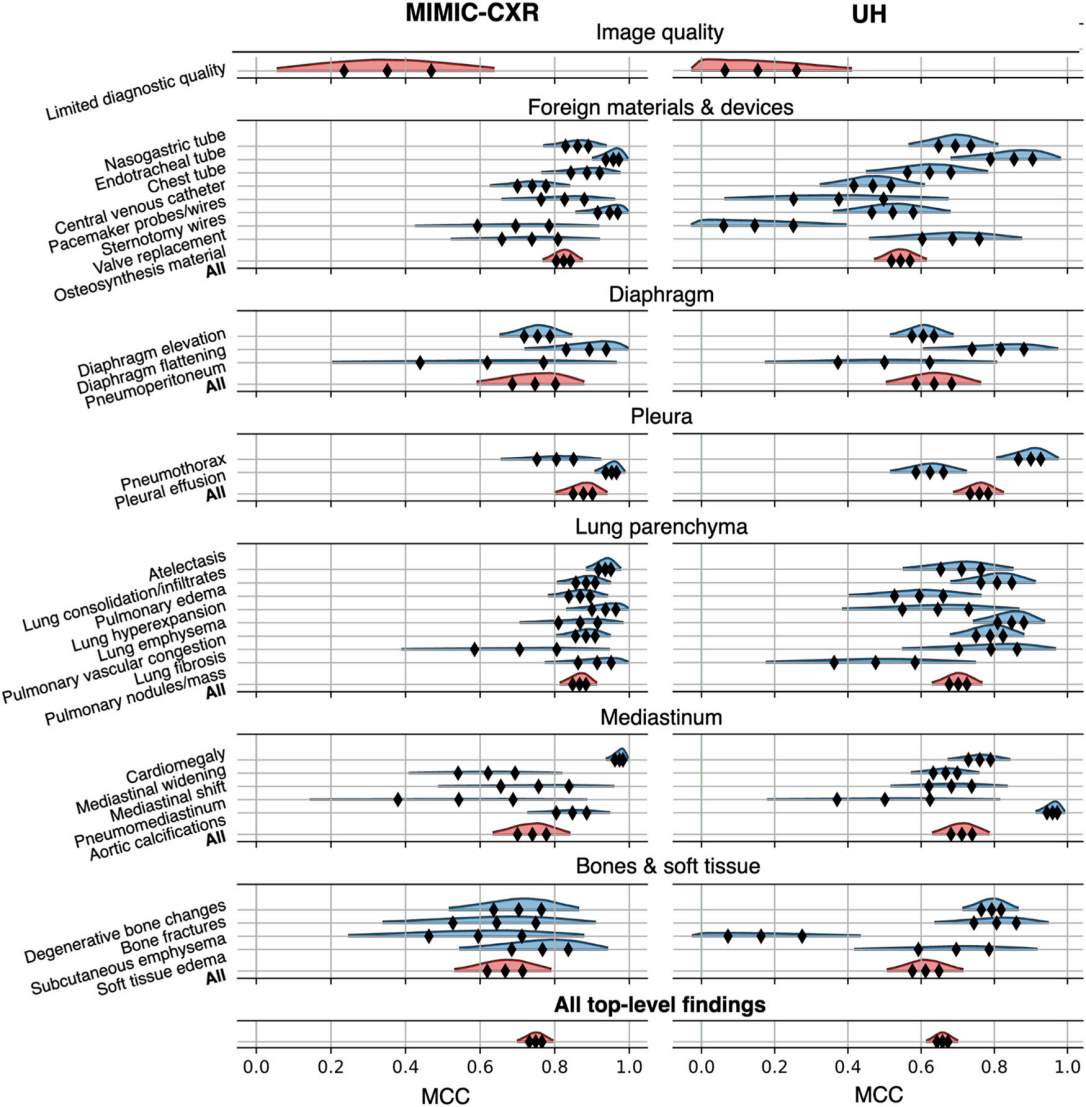
Distribution of Matthews correlation coefficient (MCC) for Llama-2-70B. The kernel density plot presents the posterior distribution of the MCC with the 94% highest density interval. Rhomboid markers denote quartiles. The red distributions represent the cumulative MCC across all findings in a template section. MIMIC-CXR, MIMIC chest X-ray cohort; UH, University Hospital cohort

The process of generating a single structured report by the LLM took between 10 and 30 s, whereas it took between 30 s and 3 min for human readers. Table 3 presents the comparative performance between Llama-2-70B and the human evaluators in the MIMIC-CXR cohort. For all top-level findings, Llama-2-70B achieved an MCC of 0.75 (94% HDI: 0.70–0.80), on par with both human readers, who reached an MCC of 0.74 (0.70–0.79), and 0.74 (0.69–0.79) respectively. Llama-2-70B achieved a sensitivity of 0.80 (0.75–0.85) and a specificity of 0.99 (0.99–0.99), closely paralleled by human readers. The results for the UH cohort are presented in Table 4. For all top-level findings, Llama-2-70B exhibited an MCC of 0.66 (0.62–0.70) and F1 score of 0.68 (94% HDI: 0.64–0.72), while Reader 1 achieved an MCC of 0.67 (0.62–0.71) and Reader 2 an MCC of 0.67 (0.62–0.70). The sensitivity was lower in this cohort: 0.75 for Llama-2-70B, 0.66 and 0.75, respectively, for human readers. Despite this, Llama-2-70B maintained a high specificity of 0.97 (0.97–0.98). Figure 3 displays detailed MCC distributions for the LLM for each finding individually and collectively by section, showing the variation in its performance. As expected with lower information, less frequent findings, such as limited diagnostic quality, pneumoperitoneum, or pneumomediastinum had wider posterior distributions in both cohorts. Analogous plots for human readers are presented in the Supplementary Figs. S1 and S2. A detailed comparative performance evaluation for every top-level finding is presented in Supplementary Tables S3 and S4.

**Table 3:** Performance metrics for the MIMIC chest X-ray (MIMIC-CXR) dataset

Template section	Metric	Llama-2-70B	Reader 1	Reader 2
Image quality	MCC	0.35 (0.05, 0.63)	0.64 (0.41, 0.85)	0.47 (0.17, 0.74)
	F1	0.32 (0.04, 0.60)	0.64 (0.41, 0.85)	0.44 (0.14, 0.73)
	Sensitivity	0.22 (0.01, 0.46)	0.78 (0.54, 0.99)	0.33 (0.08, 0.61)
	Specificity	0.97 (0.95, 0.99)	0.99 (0.98, 1.00)	0.97 (0.95, 0.99)
Foreign materials and devices	MCC	0.82 (0.77, 0.88)	0.84 (0.78, 0.89)	0.80 (0.74, 0.85)
	F1	0.83 (0.77, 0.88)	0.84 (0.79, 0.90)	0.81 (0.75, 0.86)
	Sensitivity	0.83 (0.78, 0.89)	0.85 (0.79, 0.91)	0.80 (0.74, 0.86)
	Specificity	0.98 (0.97, 0.99)	0.99 (0.98, 0.99)	0.98 (0.97, 0.98)
Diaphragm	MCC	0.74 (0.59, 0.88)	0.59 (0.44, 0.74)	0.73 (0.58, 0.86)
	F1	0.75 (0.60, 0.89)	0.57 (0.41, 0.73)	0.74 (0.59, 0.88)
	Sensitivity	0.84 (0.69, 0.98)	0.55 (0.38, 0.71)	0.82 (0.67, 0.97)
	Specificity	0.99 (0.99, 1.00)	0.95 (0.94, 0.97)	0.99 (0.98, 1.00)
Pleura	MCC	0.87 (0.80, 0.94)	0.82 (0.74, 0.91)	0.85 (0.77, 0.93)
	F1	0.89 (0.83, 0.95)	0.84 (0.76, 0.92)	0.87 (0.80, 0.94)
	Sensitivity	0.90 (0.83, 0.98)	0.80 (0.70, 0.90)	0.87 (0.78, 0.95)
	Specificity	0.98 (0.96, 0.99)	0.97 (0.95, 0.99)	0.97 (0.96, 0.99)
Lung parenchyma	MCC	0.86 (0.82, 0.91)	0.83 (0.78, 0.88)	0.84 (0.79, 0.89)
	F1	0.88 (0.83, 0.93)	0.85 (0.80, 0.90)	0.86 (0.81, 0.90)
	Sensitivity	0.91 (0.86, 0.96)	0.87 (0.81, 0.93)	0.91 (0.86, 0.95)
	Specificity	0.99 (0.98, 0.99)	0.98 (0.97, 0.98)	0.98 (0.98, 0.99)
Mediastinum	MCC	0.74 (0.63, 0.84)	0.77 (0.67, 0.87)	0.73 (0.62, 0.83)
	F1	0.74 (0.63, 0.84)	0.78 (0.67, 0.88)	0.73 (0.63, 0.84)
	Sensitivity	0.81 (0.71, 0.92)	0.74 (0.62, 0.86)	0.79 (0.68, 0.90)
	Specificity	0.99 (0.99, 1.00)	0.98 (0.97, 0.99)	0.99 (0.98, 0.99)
Bones and soft tissue	MCC	0.66 (0.54, 0.79)	0.71 (0.59, 0.82)	0.68 (0.55, 0.80)
	F1	0.67 (0.54, 0.80)	0.70 (0.58, 0.83)	0.67 (0.54, 0.80)
	Sensitivity	0.70 (0.55, 0.83)	0.71 (0.58, 0.84)	0.68 (0.54, 0.82)
	Specificity	0.99 (0.98, 0.99)	0.99 (0.98, 0.99)	0.99 (0.98, 0.99)
**All top-level findings**	MCC	0.75 (0.70, 0.80)	0.74 (0.70, 0.79)	0.74 (0.69, 0.79)
	F1	0.75 (0.70, 0.80)	0.74 (0.70, 0.79)	0.75 (0.70, 0.79)
	Sensitivity	0.80 (0.75, 0.85)	0.75 (0.70, 0.81)	0.79 (0.74, 0.84)
	Specificity	0.99 (0.99, 0.99)	0.98 (0.97, 0.98)	0.98 (0.98, 0.99)

Results are reported as micro-averaged mean and 94% highest density interval, aggregated by template section and across all top-level findings

**Table 4:** Performance metrics for the University Hospital (UH) dataset

Template section	Metric	Llama-2-70B	Reader 1	Reader 2
Image quality	MCC	0.17 (−0.03, 0.41)	0.83 (0.68, 0.96)	0.35 (0.09, 0.61)
	F1	0.13 (0.00, 0.34)	0.83 (0.68, 0.97)	0.34 (0.09, 0.59)
	Sensitivity	0.08 (0.00, 0.23)	0.92 (0.77, 1.00)	0.25 (0.04, 0.47)
	Specificity	0.95 (0.92, 0.98)	0.99 (0.99, 1.00)	0.96 (0.93, 0.98)
Foreign materials and devices	MCC	0.54 (0.47, 0.61)	0.72 (0.66, 0.78)	0.62 (0.56, 0.69)
	F1	0.53 (0.46, 0.60)	0.73 (0.67, 0.79)	0.64 (0.57, 0.70)
	Sensitivity	0.52 (0.46, 0.58)	0.82 (0.76, 0.88)	0.69 (0.63, 0.76)
	Specificity	0.95 (0.94, 0.96)	0.98 (0.98, 0.99)	0.97 (0.96, 0.98)
Diaphragm	MCC	0.63 (0.51, 0.76)	0.56 (0.42, 0.70)	0.63 (0.50, 0.76)
	F1	0.67 (0.54, 0.80)	0.56 (0.41, 0.70)	0.67 (0.54, 0.80)
	Sensitivity	0.81 (0.68, 0.95)	0.47 (0.32, 0.63)	0.81 (0.68, 0.95)
	Specificity	0.99 (0.98, 1.00)	0.91 (0.89, 0.93)	0.99 (0.98, 1.00)
Pleura	MCC	0.76 (0.69, 0.82)	0.82 (0.76, 0.87)	0.75 (0.69, 0.82)
	F1	0.84 (0.78, 0.89)	0.89 (0.84, 0.93)	0.84 (0.79, 0.89)
	Sensitivity	0.82 (0.75, 0.88)	0.90 (0.84, 0.95)	0.83 (0.77, 0.90)
	Specificity	0.90 (0.86, 0.93)	0.94 (0.92, 0.97)	0.90 (0.87, 0.94)
Lung parenchyma	MCC	0.70 (0.63, 0.77)	0.65 (0.58, 0.72)	0.68 (0.61, 0.75)
	F1	0.70 (0.64, 0.77)	0.65 (0.58, 0.73)	0.69 (0.62, 0.76)
	Sensitivity	0.77 (0.70, 0.84)	0.62 (0.54, 0.69)	0.76 (0.69, 0.83)
	Specificity	0.98 (0.97, 0.99)	0.97 (0.97, 0.98)	0.98 (0.97, 0.99)
Mediastinum	MCC	0.71 (0.63, 0.79)	0.73 (0.65, 0.81)	0.71 (0.63, 0.78)
	F1	0.75 (0.68, 0.83)	0.74 (0.66, 0.82)	0.75 (0.67, 0.82)
	Sensitivity	0.87 (0.79, 0.95)	0.67 (0.57, 0.76)	0.86 (0.78, 0.94)
	Specificity	0.98 (0.97, 0.99)	0.94 (0.92, 0.95)	0.97 (0.96, 0.98)
Bones and soft tissue	MCC	0.61 (0.51, 0.72)	0.65 (0.55, 0.75)	0.62 (0.51, 0.72)
	F1	0.62 (0.52, 0.72)	0.65 (0.55, 0.75)	0.62 (0.52, 0.72)
	Sensitivity	0.67 (0.57, 0.76)	0.69 (0.60, 0.79)	0.65 (0.55, 0.74)
	Specificity	0.98 (0.96, 0.99)	0.97 (0.96, 0.98)	0.98 (0.97, 0.99)
**All top-level findings**	MCC	0.66 (0.62, 0.70)	0.67 (0.62, 0.71)	0.66 (0.62, 0.70)
	F1	0.68 (0.64, 0.72)	0.68 (0.63, 0.72)	0.68 (0.64, 0.72)
	Sensitivity	0.75 (0.71, 0.79)	0.66 (0.61, 0.70)	0.75 (0.71, 0.79)
	Specificity	0.97 (0.97, 0.98)	0.96 (0.95, 0.96)	0.97 (0.97, 0.98)

Results are reported as micro-averaged mean and 94% highest density interval, aggregated by template sections

### Inter-rater differences

**Fig. 4:**
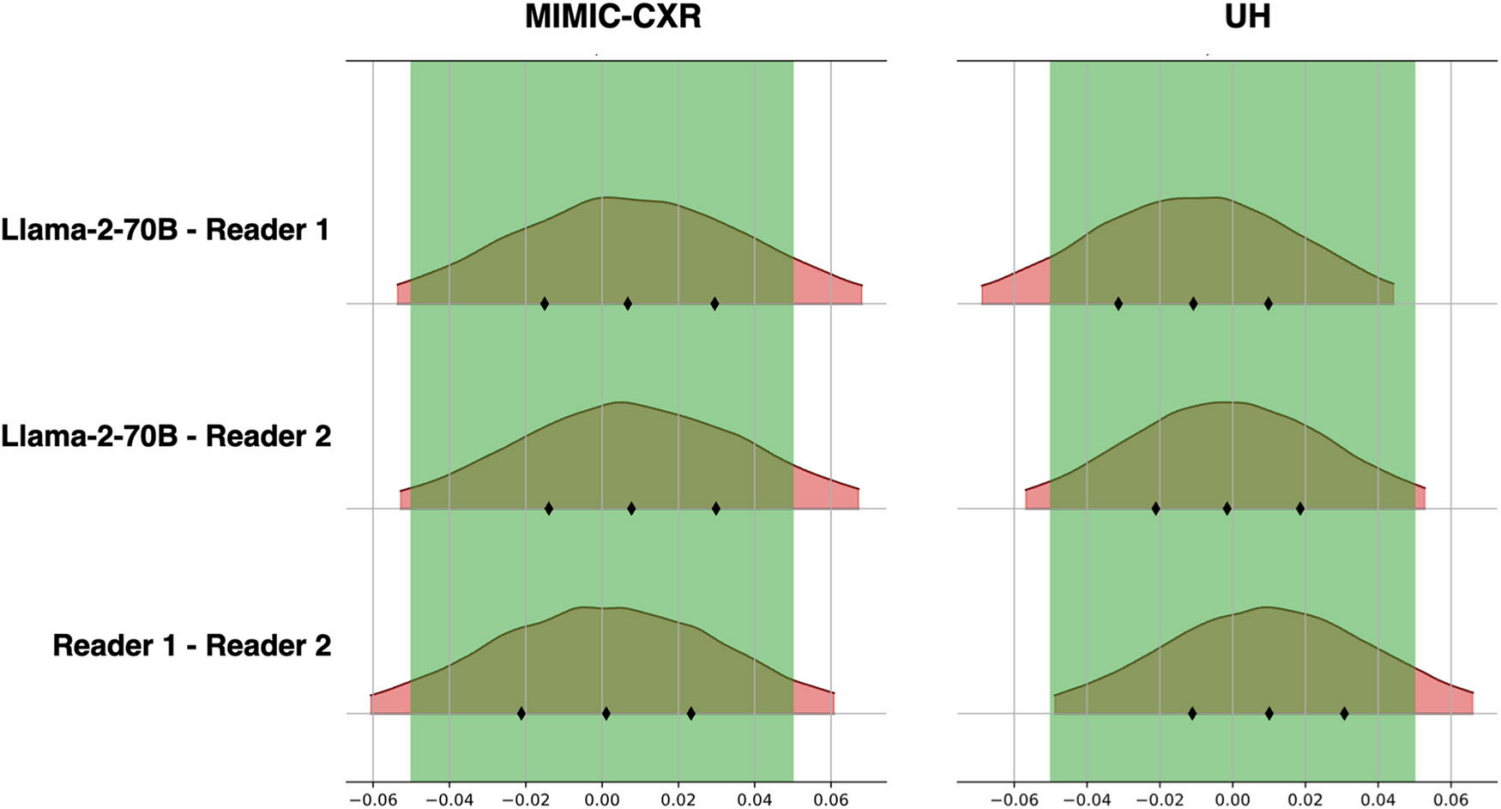
Distribution of pairwise differences in Matthews correlation coefficient (MCC). The kernel density plot shows the posterior distribution of the MCC pairwise differences with the 94% highest density interval. Rhomboid markers denote quartiles. The green vertical shaded area is the region of practical equivalence (−0.05, 0.05). The red distributions represent the cumulative differences across all labels. MIMIC-CXR, MIMIC chest X-ray cohort; UH, University Hospital cohort

Table 5 summarizes inter-rater differences across the MIMIC-CXR and UH cohorts regarding micro-averaged pairwise differences. The cumulative distributions of pairwise differences in MCC between readers are shown in Fig. 4. Aggregated across all top-level findings, the probability distributions of the MCC difference between the LLM and humans exceeded the bounds of the ROPE on both sides for the English and on the left-hand side for German reports. For MIMIC-CXR, the results were as follows: LLM - Reader 1: 0.01 (94% HDI: −0.05 to 0.07), LLM - Reader 2: 0.01 (−0.05 to 0.07), Reader 1 - Reader 2: 0.00 (−0.06 to 0.06). For the UH cohort, the delta distributions were: LLM - Reader 1: −0.01 (−0.07 to 0.05), LLM - Reader 2: 0.00 (−0.06 to 0.06), Reader 1 - Reader 2: 0.01 (−0.0 to 0.07). Those results indicated that, with a high probability, the LLM was non-inferior to human readers. A detailed pairwise evaluation of the inter-rater differences is presented in Supplementary Figs. S5–S7.

**Table 5:** Inter-rater differences

	MIMIC-CXR			
Template section	Metric	LLM - Reader 1	LLM - Reader 2	Reader 1 - Reader 2
Image quality	ΔMCC	−0.28 (−0.65, 0.08)	−0.12 (−0.53, 0.29)	0.17 (−0.19, 0.52)
	ΔF1	−0.33 (−0.68, 0.04)	−0.13 (−0.56, 0.29)	0.20 (−0.17, 0.57)
Foreign materials and devices	ΔMCC	−0.01 (−0.09, 0.06)	0.02 (−0.05, 0.09)	0.04 (−0.04, 0.11)
	ΔF1	−0.02 (−0.09, 0.06)	0.02 (−0.05, 0.10)	0.04 (−0.04, 0.11)
Diaphragm	ΔMCC	0.15 (−0.06, 0.35)	0.01 (−0.18, 0.21)	−0.13 (−0.33, 0.07)
	ΔF1	0.17 (−0.04, 0.39)	0.01 (−0.19, 0.21)	−0.16 (−0.37, 0.05)
Pleural cavity	ΔMCC	0.05 (−0.06, 0.16)	0.02 (−0.08, 0.12)	−0.03 (−0.14, 0.09)
	ΔF1	0.05 (−0.06, 0.15)	0.02 (−0.08, 0.11)	−0.03 (−0.14, 0.07)
Lung parenchyma	ΔMCC	0.03 (−0.03, 0.10)	0.03 (−0.04, 0.09)	−0.01 (−0.07, 0.06)
	ΔF1	0.03 (−0.03, 0.10)	0.02 (−0.04, 0.09)	−0.01 (−0.07, 0.05)
Mediastinum	ΔMCC	−0.03 (−0.18, 0.10)	0.01 (−0.13, 0.15)	0.05 (−0.10, 0.19)
	ΔF1	−0.04 (−0.18, 0.10)	0.01 (−0.13, 0.15)	0.05 (−0.09, 0.20)
Bones and soft tissue	ΔMCC	−0.04 (−0.21, 0.13)	−0.01 (−0.19, 0.16)	0.03 (−0.14, 0.20)
	ΔF1	−0.04 (−0.21, 0.14)	−0.01 (−0.19, 0.17)	0.03 (−0.14, 0.21)
**All top-level findings**	ΔMCC	0.01 (−0.05, 0.07)	0.01 (−0.05, 0.07)	0.00 (−0.06, 0.06)
	ΔF1	0.01 (−0.05, 0.07)	0.01 (−0.06, 0.07)	0.00 (−0.06, 0.06)
	**UH**			
Image quality	ΔMCC	−0.65 (−0.93, −0.37)	−0.18 (−0.53, 0.19)	0.48 (0.18, 0.77)
	ΔF1	−0.70 (−0.93, −0.43)	−0.20 (−0.54, 0.14)	0.50 (0.20, 0.78)
Foreign materials and devices	ΔMCC	−0.18 (−0.26, −0.08)	−0.08 (−0.17, 0.01)	0.10 (0.01, 0.18)
	ΔF1	−0.20 (−0.29, −0.10)	−0.11 (−0.19, 0.01)	0.09 (0.01, 0.17)
Diaphragm	ΔMCC	0.07 (−0.12, 0.26)	0.00 (−0.17, 0.17)	−0.07 (−0.26, 0.12)
	ΔF1	0.12 (−0.07, 0.31)	0.00 (−0.17, 0.17)	−0.12 (−0.31, 0.07)
Pleural cavity	ΔMCC	−0.06 (−0.15, 0.03)	0.01 (−0.09, 0.10)	0.07 (−0.02, 0.16)
	ΔF1	−0.05 (−0.12, 0.01)	0.00 (−0.07, 0.07)	0.05 (−0.02, 0.11)
Lung parenchyma	ΔMCC	0.05 (−0.05, 0.14)	0.02 (−0.07, 0.11)	−0.03 (−0.13, 0.06)
	ΔF1	0.05 (−0.05, 0.15)	0.02 (−0.07, 0.10)	−0.03 (−0.13, 0.07)
Mediastinum	ΔMCC	−0.02 (−0.13, 0.09)	0.00 (−0.10, 0.10)	0.02 (−0.08, 0.14)
	ΔF1	0.01 (−0.10, 0.12)	0.00 (−0.09, 0.10)	−0.01 (−0.11, 0.11)
Bones and soft tissue	ΔMCC	−0.04 (−0.18, 0.10)	0.00 (−0.14, 0.14)	0.03 (−0.11, 0.18)
	ΔF1	−0.02 (−0.16, 0.11)	0.00 (−0.13, 0.13)	0.03 (−0.11, 0.17)
**All top-level findings**	ΔMCC	−0.01 (−0.07, 0.05)	0.00 (−0.06, 0.06)	0.01 (−0.05, 0.07)
	ΔF1	0.00 (−0.05, 0.06)	0.00 (−0.06, 0.05)	−0.01 (−0.06, 0.05)

Pairwise differences (Δ) are reported as mean and 94% highest density interval of the MCC distributions, for template sections and averaged across all top-level findings

*MCC* Matthews correlation coefficient, *MIMIC-CXR* MIMIC chest X-ray cohort, *UH* University Hospital cohort

## Discussion

In this study, we developed and evaluated a novel method using a locally hosted, open-source large language model, Llama-2-70B, to convert clinical free-text reports into template-based, machine-readable structured reports. Structured reporting offers many advantages, yet its adoption in clinical practice remains limited. An LLM-based approach could enable radiologists to preserve their traditional narrative reporting style. Our approach guarantees the creation of valid, fully structured reports in all instances. The LLM achieved mean Matthews correlation coefficients of 0.75 (94% HDI: 0.70–0.80) within the MIMIC-CXR cohort and 0.68 (94% HDI: 0.64–0.72) within the UH cohort. Our results show evidence (majority of probability density inside the ROPEs) for parity with human readers in both cohorts.

Interestingly, both LLM and human readers achieved a high specificity of over 0.95, and lower sensitivity ranging from 0.66 to 0.80. This result can primarily be attributed to the imbalance between positive and negative findings in the reports. However, a lower sensitivity poses a risk of missing critical findings and suggests room for improvement.

The MCCs for German reports were noticeably lower than for English reports. Although German was the second most common language in Llama-2’s training corpus, it constituted only 0.17% of the dataset, with English covering 89.7% [9]. On top of that, German reports sometimes included abbreviations differing in meaning from their English counterparts. For instance, PE typically stands for pulmonary embolism in English but for “Pleuraerguss” (pleural effusion) in German. Another challenge encountered is the vague wording of free-text reports, which use terms such as “small to moderate,” “possible,” and “cannot be excluded,” which cannot be unequivocally mapped against a fully structured template. The problem was more pronounced for the German reports. It could also explain lower MCC scores for human readers in the UH cohort. Hallucinations, where the LLM produces factually incorrect or nonsensical outputs, are a significant concern for clinical applications. We observed the LLM sometimes incorrectly predicted the presence of pleural drainage catheters when they were not explicitly mentioned in the report but were plausible given other findings such as pneumonia and large pleural effusion. Further research should prospectively evaluate the performance of LLMs for structured reporting when the reporting radiologist is aware of the template and its items. Other potential performance improvements include developing feedback loops for continual learning from radiologists’ corrections and curating guidelines for interpreting vague language to refine prompts. Additionally, integrating multi-modal learning, where the LLM can access both textual reports and associated medical images, could enhance its ability to cross-reference findings and improve accuracy.

Automatic retrospective structuring of radiology reports within an institution has many potential applications. It can support research, enabling faster retrieval of relevant cases for a query and automatic label extraction for imaging-based AI algorithms. It can simplify longitudinal patient monitoring and can be used to extract information from trial reports, e.g., with RECIST or Lugano criteria [19]. Finally, it may help in communication with other specialties and non-clinical researchers. On the other hand, retrospectively creating a fully structured report from a free-text report might lead to some information loss, typically due to two reasons: (1) the free-text report including a finding not covered by the template, and (2) a free-text finding being ambiguous or impossible to map to the options specified in the template unequivocally.

Several recent publications have explored the post-hoc structuring of radiology reports using LLMs. Adams et al [11] devised a two-step approach: GPT-4 automatically selected an appropriate template and then created a structured report. Mukherjee et al [20] used single- and multi-step prompting of a locally hosted Vicuna-13B model to extract 13 binary findings from chest radiography reports. Our approach has a few important advantages over the ones mentioned above. First, thanks to the constrained generation and limiting the LLM inference to predefined answers, structuring is immune to adversarial attacks, including prompt injection, which could be attempted to extract sensitive information [21]. Second, using a local model ensures that data does not leave the institution, satisfying data protection regulations. Finally, we evaluated a fine-grained and fully structured template, and a dataset of internal, real-world non-English reports.

The most advanced LLMs are primarily accessible through cloud-based services, offering access through graphical or application programming interfaces (APIs). These models remain a cost-effective and scalable solution for scenarios where data protection is not a primary concern. However, the difference in performance and speed between them and their open-access alternatives is becoming less significant. For instance, in a recent study, Llama-2-70B achieved an accuracy of 63.8% on USMLE-style questions from the MedQA dataset, a higher score than that of GPT-3.5, which was the state-of-the-art commercial model upon its release 9 months before Llama-2 [22]. In comparison, human experts scored 87%. Several fine-tuned models have been derived from Llama-2, including Vicuna, Alpaca, and other model families [20]. Llama-2 models were also successfully fine-tuned on medicine-specific data sources, demonstrating a moderate performance improvement in medical reasoning tasks over the base models [22].

Local, open-source LLMs offer the user full control over the model and eliminate the risk of its discontinuation by an external provider. A rich open-source software ecosystem simplifies switching between models and setups, including widely adopted libraries like *Huggingface*, *LangChain*, and *Guidance*. Although the computing requirements are still a limitation to running LLMs locally, smaller LLMs like *Mistral 7B*, which can run on consumer-grade machines, are showing performance improvements [23]. Further, by applying a technique called quantization, which reduces the number of bits in the model’s weights [24], LLMs become faster and incur lower computational and memory costs. This technique was also used in our study. To improve sensitivity and reduce the risk of missing critical findings, strategies such as domain-specific fine-tuning on medical datasets, prompt optimization, and optimizing template wording can be implemented. Finally, ensuring radiologists are aware of and include all required information in their reports can significantly enhance performance.

As with all non-experimental studies, the ability to draw causal conclusions is limited. Results should hence be interpreted as hypothesis-generating. Our study was performed on a relatively small sample size, and more data would be needed to make binary decisions in the Bayesian ROPE framework. Consequently, the effect size is expected to remain small within our cohorts. Further, defining the reference standard was challenging due to the rigidity of templates and the ambiguity of retrospectively collected free-text reports. Our evaluation focused on a single modality and anatomic region, and we only evaluated the performance of top-level findings. Statistics on nested elements are subject to conditional probabilities lying beyond the scope of this manuscript. Finally, we assessed only one prompt, and the LLM was not fine-tuned to medical and German contexts. Potential improvements by prompt engineering, model fine-tuning, and preprocessing of reports using machine translation remain subject to future research. To enhance the reliability of LLM-generated reports, future research could leverage Bayesian techniques, model ensembling, and Monte Carlo Dropout to quantify and improve confidence estimates.

In conclusion, on-premise, open-source LLMs are able to automatically structure free-text radiology reports using a fine-grained template with approximately human-level accuracy, highlighting their potential to significantly enhance the efficiency and reliability of processing and interpreting clinical reports. However, the understanding of semantics varied across languages and imaging findings. Further evaluations in larger clinical cohorts are needed to establish their usefulness in the clinical setting.

## Supplementary information

Supplement

## Abbreviations

CXR: Chest radiograph
GPT: Generative Pre-trained Transformer
LLM: Large language model
MCC: Matthews correlation coefficient
MIMIC-CXR: MIMIC chest X-ray cohort
ROPE: Region of practical equivalence
UH: University Hospital cohort
