# Supplementary material for: Automatic structuring of radiology reports with on-premise open-source large language models
Source: Eur Radiol. 2024 Oct 10;35(4):2018–29. doi: 10.1007/s00330-024-11074-y (PMC11913902; doi:10.1007/s00330-024-11074-y)
Supplement: Supplementary file 1 — Supplement [file 330_2024_11074_MOESM1_ESM.pdf]

Supplement

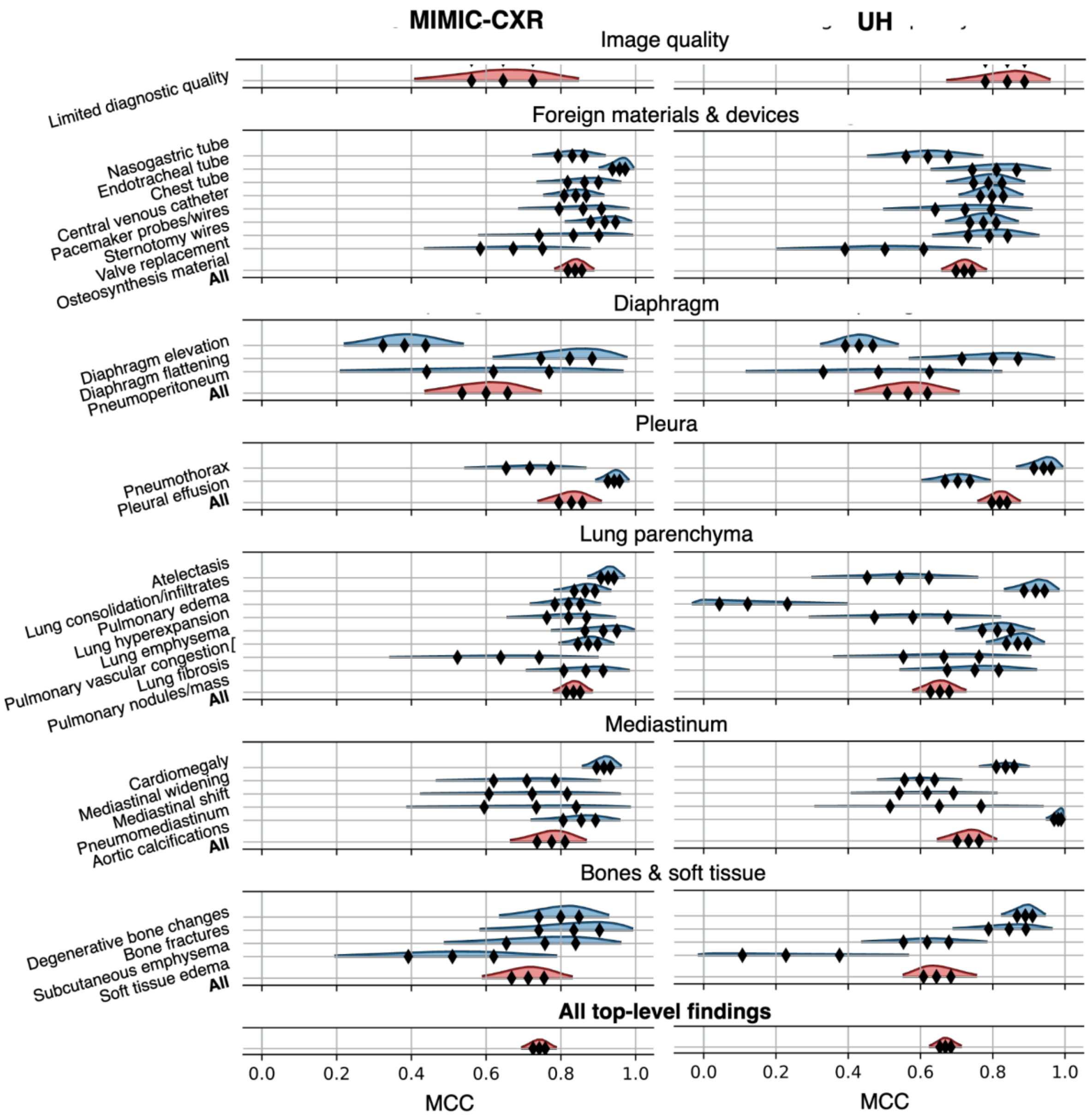

Figure S1. Distribution of Matthews correlation coefficient (MCC) for Reader 1. The kernel density plot presents the posterior distribution of the MCC with the 94% highest density interval. Rhomboid markers denote quartiles. The red distributions represent the cumulative MCC across all findings in a template section. *MIMIC-CXR - MIMIC Chest X-ray cohort, UH - University Hospital cohort*

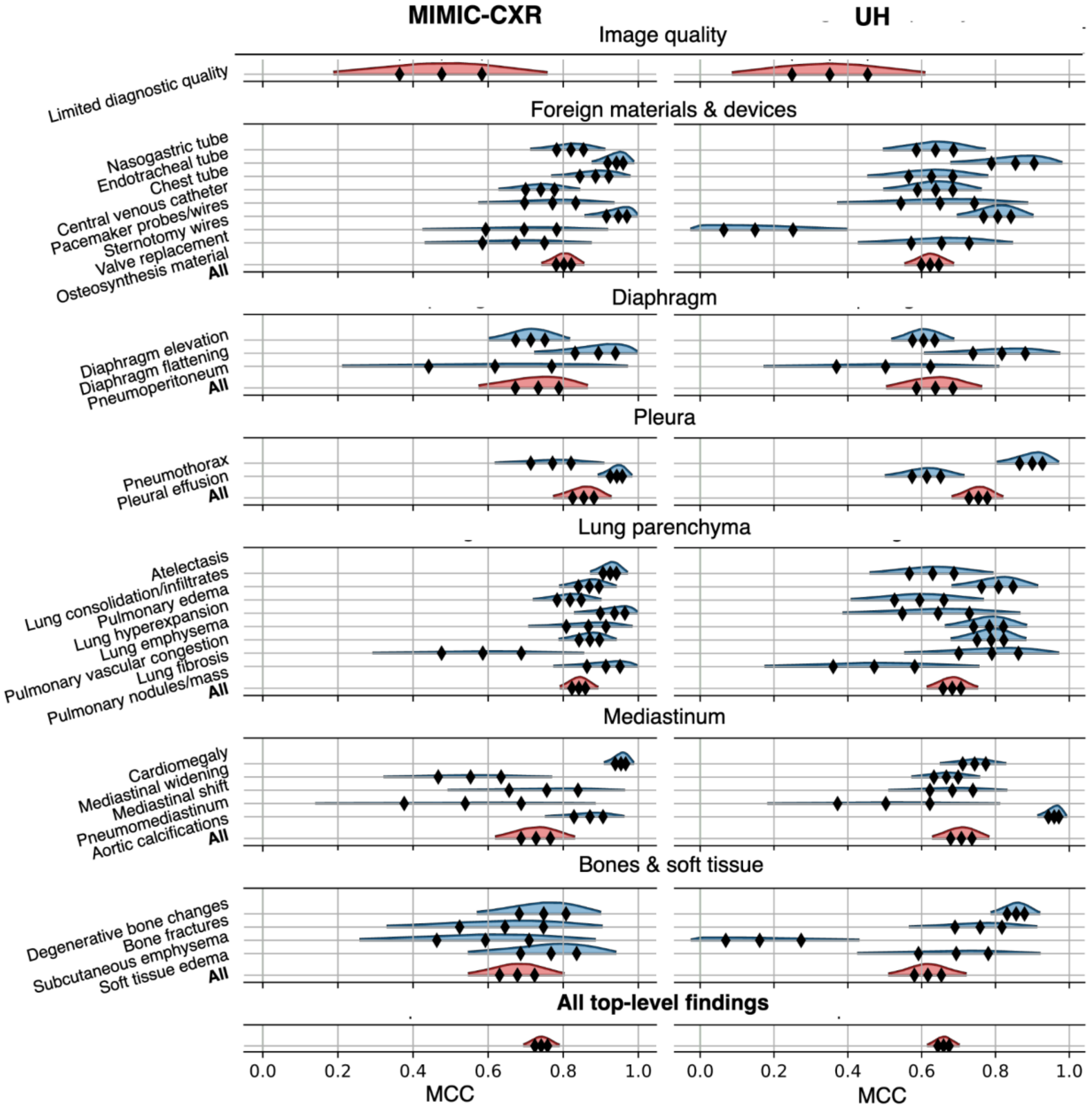

Figure S2. Distribution of Matthews correlation coefficient (MCC) for Reader 2. The kernel density plot presents the posterior distribution of the MCC with the 94% highest density interval. Rhomboid markers denote quartiles. The red distributions represent the cumulative MCC across all findings in a template section. *MIMIC-CXR - MIMIC Chest X-ray cohort, UH - University Hospital cohort*

Table S3. Detailed performance for each top-level finding in the MIMIC Chest X-ray (MIMIC-CXR) cohort. Results are reported as micro-averaged mean and 94% highest density interval. *MCC - Matthews correlation coefficient*

Finding	Metric	Reader 1	Reader 2	Llama-2-70B
limited diagnostic quality	MCC	0.64 (0.41;0.85)	0.47 (0.18;0.75)	0.35 (0.06;0.63)
	F1	0.38 (0.22;0.54)	0.71 (0.60;0.81)	0.75 (0.66;0.84)
	Sensitivity	0.81 (0.62;0.98)	0.88 (0.72;0.99)	0.88 (0.72;1.00)
	Specificity	0.60 (0.20;0.96)	0.60 (0.21;0.97)	0.60 (0.20;0.96)
diaphragm elevation	MCC	0.71 (0.54;0.87)	0.76 (0.61;0.90)	0.80 (0.66;0.93)
	F1	0.94 (0.90;0.98)	0.94 (0.90;0.98)	0.95 (0.91;0.99)
	Sensitivity	0.92 (0.87;0.97)	0.92 (0.87;0.97)	0.93 (0.89;0.98)
	Specificity	0.86 (0.79;0.94)	0.87 (0.79;0.94)	0.88 (0.81;0.95)
diaphragm flattening	MCC	0.82 (0.72;0.91)	0.81 (0.72;0.90)	0.86 (0.78;0.94)
	F1	0.81 (0.66;0.95)	0.93 (0.83;1.00)	0.93 (0.83;1.00)
	Sensitivity	0.90 (0.77;1.00)	0.85 (0.71;0.98)	0.85 (0.70;0.98)
	Specificity	0.87 (0.80;0.94)	0.87 (0.79;0.94)	0.88 (0.81;0.95)
pneumoperitoneum	MCC	0.62 (0.33;0.89)	0.58 (0.28;0.84)	0.69 (0.40;0.95)
	F1	0.85 (0.71;0.98)	0.90 (0.78;1.00)	0.90 (0.78;1.00)
	Sensitivity	0.91 (0.86;0.96)	0.95 (0.91;0.99)	0.97 (0.94;1.00)
	Specificity	0.70 (0.47;0.91)	0.55 (0.32;0.77)	0.61 (0.41;0.81)
pneumothorax	MCC	0.70 (0.42;0.95)	0.74 (0.49;0.96)	0.74 (0.49;0.96)
	F1	0.71 (0.39;0.98)	0.53 (0.15;0.89)	0.53 (0.15;0.89)
	Sensitivity	0.84 (0.72;0.96)	0.86 (0.75;0.96)	0.84 (0.73;0.95)
	Specificity	0.79 (0.64;0.93)	0.74 (0.57;0.90)	0.70 (0.52;0.86)
pleural effusion	MCC	0.81 (0.58;0.99)	0.63 (0.33;0.90)	0.63 (0.33;0.90)
	F1	0.74 (0.49;0.96)	0.58 (0.26;0.89)	0.58 (0.25;0.87)
	Sensitivity	0.50 (0.20;0.79)	0.75 (0.54;0.94)	0.75 (0.55;0.94)
	Specificity	0.38 (-0.00;0.81)	0.38 (-0.00;0.81)	0.38 (-0.00;0.81)
atelectasis	MCC	0.74 (0.70;0.79)	0.74 (0.69;0.79)	0.75 (0.70;0.79)
	F1	0.64 (0.40;0.85)	0.44 (0.15;0.74)	0.31 (0.05;0.60)
	Sensitivity	0.33 (0.16;0.50)	0.75 (0.65;0.85)	0.78 (0.68;0.87)
	Specificity	0.81 (0.61;0.98)	0.88 (0.72;0.99)	0.88 (0.72;1.00)
lung consolidation/infiltrates	MCC	0.58 (0.17;0.96)	0.58 (0.18;0.97)	0.58 (0.17;0.96)
	F1	0.72 (0.55;0.88)	0.78 (0.64;0.91)	0.81 (0.69;0.93)
	Sensitivity	0.96 (0.94;0.99)	0.96 (0.94;0.99)	0.97 (0.94;0.99)
	Specificity	0.96 (0.94;0.99)	0.96 (0.94;0.98)	0.97 (0.94;0.99)

pulmonary edema	MCC	0.89 (0.82;0.95)	0.90 (0.83;0.95)	0.91 (0.85;0.96)
	F1	0.85 (0.77;0.93)	0.85 (0.77;0.92)	0.89 (0.82;0.95)
	Sensitivity	0.82 (0.67;0.95)	0.93 (0.84;1.00)	0.93 (0.84;1.00)
	Specificity	0.90 (0.78;1.00)	0.86 (0.71;0.98)	0.86 (0.71;0.98)
lung hyperexpansion	MCC	0.90 (0.84;0.95)	0.90 (0.84;0.96)	0.91 (0.85;0.96)
	F1	0.61 (0.30;0.90)	0.55 (0.25;0.85)	0.68 (0.38;0.95)
	Sensitivity	0.86 (0.71;0.98)	0.90 (0.78;1.00)	0.90 (0.78;1.00)
	Specificity	0.95 (0.92;0.98)	0.97 (0.95;0.99)	0.98 (0.97;1.00)
lung emphysema	MCC	0.69 (0.46;0.91)	0.56 (0.33;0.77)	0.61 (0.40;0.81)
	F1	0.70 (0.41;0.96)	0.73 (0.47;0.96)	0.73 (0.48;0.96)
	Sensitivity	0.70 (0.36;0.98)	0.52 (0.13;0.88)	0.52 (0.12;0.87)
	Specificity	0.85 (0.73;0.96)	0.87 (0.76;0.96)	0.85 (0.74;0.95)
pulmonary vascular congestion/volume overload	MCC	0.80 (0.66;0.94)	0.75 (0.58;0.91)	0.71 (0.55;0.87)
	F1	0.81 (0.57;0.99)	0.63 (0.33;0.91)	0.63 (0.33;0.90)
	Sensitivity	0.73 (0.48;0.96)	0.57 (0.24;0.89)	0.57 (0.23;0.87)
	Specificity	0.48 (0.17;0.78)	0.75 (0.54;0.95)	0.75 (0.53;0.95)
lung fibrosis	MCC	0.35 (0.00;0.80)	0.36 (0.00;0.80)	0.36 (0.00;0.81)
	F1	0.74 (0.70;0.79)	0.75 (0.70;0.79)	0.75 (0.70;0.80)
	Sensitivity	0.78 (0.53;0.99)	0.33 (0.07;0.60)	0.22 (0.02;0.46)
	Specificity	0.21 (0.08;0.33)	0.91 (0.82;0.99)	0.97 (0.92;1.00)
pulmonary nodules/mass	MCC	0.78 (0.54;0.99)	0.89 (0.70;1.00)	0.89 (0.70;1.00)
	F1	0.67 (0.25;1.00)	0.67 (0.24;1.00)	0.67 (0.25;1.00)
	Sensitivity	0.63 (0.43;0.83)	0.79 (0.62;0.95)	0.84 (0.69;0.98)
	Specificity	0.96 (0.93;1.00)	0.95 (0.91;0.99)	0.96 (0.93;0.99)
cardiomegaly	MCC	0.94 (0.90;0.98)	0.96 (0.93;0.99)	0.98 (0.96;1.00)
	F1	0.81 (0.71;0.92)	0.92 (0.84;0.98)	0.94 (0.87;0.99)
	Sensitivity	0.88 (0.78;0.96)	0.93 (0.85;0.99)	0.93 (0.85;0.99)
	Specificity	0.80 (0.61;0.97)	0.93 (0.82;1.00)	0.93 (0.82;1.00)
mediastinal widening	MCC	0.91 (0.75;1.00)	0.91 (0.75;1.00)	0.91 (0.76;1.00)
	F1	0.94 (0.88;0.99)	0.92 (0.85;0.98)	0.92 (0.85;0.98)
	Sensitivity	0.80 (0.49;1.00)	0.80 (0.50;1.00)	0.80 (0.49;1.00)
	Specificity	0.91 (0.76;1.00)	0.91 (0.76;1.00)	0.91 (0.76;1.00)
mediastinal shift	MCC	0.91 (0.85;0.96)	0.96 (0.92;0.99)	0.99 (0.97;1.00)
	F1	0.60 (0.33;0.87)	0.70 (0.44;0.94)	0.80 (0.58;0.99)
	Sensitivity	0.67 (0.35;0.97)	0.83 (0.57;1.00)	0.83 (0.57;1.00)

	Specificity	0.75 (0.40;1.00)	0.50 (0.11;0.90)	0.50 (0.10;0.89)
pneumomediastinum	MCC	0.79 (0.62;0.95)	0.95 (0.85;1.00)	0.95 (0.85;1.00)
	F1	0.81 (0.64;0.97)	0.69 (0.48;0.90)	0.75 (0.56;0.93)
	Sensitivity	0.83 (0.57;1.00)	0.67 (0.34;0.97)	0.67 (0.35;0.97)
	Specificity	0.83 (0.57;1.00)	0.50 (0.15;0.84)	0.50 (0.15;0.84)
aortic calcifications	MCC	0.38 (0.09;0.67)	0.88 (0.66;1.00)	0.87 (0.67;1.00)
	F1	0.50 (0.04;0.98)	0.50 (0.01;0.95)	0.50 (0.02;0.96)
	Sensitivity	0.75 (0.70;0.80)	0.79 (0.74;0.84)	0.80 (0.75;0.85)
	Specificity	0.99 (0.98;1.00)	0.97 (0.95;0.99)	0.97 (0.95;0.99)
degenerative bone changes	MCC	0.87 (0.82;0.91)	0.98 (0.96;1.00)	0.99 (0.98;1.00)
	F1	0.99 (0.98;1.00)	0.99 (0.99;1.00)	0.99 (0.99;1.00)
	Sensitivity	1.00 (0.99;1.00)	1.00 (0.99;1.00)	1.00 (0.99;1.00)
	Specificity	0.96 (0.94;0.99)	0.98 (0.96;1.00)	0.98 (0.97;1.00)
bone fractures	MCC	0.98 (0.95;1.00)	0.97 (0.94;0.99)	0.98 (0.95;1.00)
	F1	0.94 (0.90;0.98)	0.96 (0.93;0.99)	0.98 (0.95;1.00)
	Sensitivity	0.95 (0.91;0.98)	0.97 (0.95;0.99)	0.98 (0.96;1.00)
	Specificity	0.97 (0.94;0.99)	0.98 (0.96;1.00)	0.98 (0.96;1.00)
subcutaneous emphysema	MCC	0.98 (0.97;1.00)	0.99 (0.99;1.00)	0.99 (0.99;1.00)
	F1	0.99 (0.99;1.00)	0.99 (0.99;1.00)	0.99 (0.99;1.00)
	Sensitivity	0.98 (0.96;1.00)	0.97 (0.95;0.99)	0.97 (0.95;0.99)
	Specificity	1.00 (0.99;1.00)	1.00 (0.99;1.00)	1.00 (0.99;1.00)
soft tissue edema	MCC	0.99 (0.99;1.00)	0.99 (0.99;1.00)	0.99 (0.99;1.00)
	F1	0.94 (0.89;0.97)	0.97 (0.94;0.99)	0.99 (0.98;1.00)
	Sensitivity	0.98 (0.96;1.00)	0.99 (0.97;1.00)	0.99 (0.98;1.00)
	Specificity	0.99 (0.98;1.00)	1.00 (0.99;1.00)	1.00 (0.99;1.00)
pneumothorax	MCC	1.00 (0.99;1.00)	0.99 (0.98;1.00)	0.99 (0.98;1.00)
	F1	0.98 (0.96;1.00)	0.99 (0.98;1.00)	0.99 (0.98;1.00)
	Sensitivity	0.98 (0.97;1.00)	0.97 (0.95;0.99)	0.98 (0.96;1.00)
	Specificity	1.00 (0.99;1.00)	0.99 (0.98;1.00)	0.99 (0.98;1.00)
**All top-level findings**	MCC	1.00 (0.99;1.00)	0.99 (0.97;1.00)	0.99 (0.97;1.00)
	F1	0.98 (0.96;0.99)	0.99 (0.99;1.00)	0.99 (0.99;1.00)
	Sensitivity	1.00 (0.99;1.00)	1.00 (0.99;1.00)	1.00 (0.99;1.00)
	Specificity	0.98 (0.97;0.98)	0.98 (0.98;0.99)	0.99 (0.99;0.99)

Table S4. Detailed performance for each top-level finding in the University Hospital (UH) cohort. Results are reported as micro-averaged mean and 94% highest density interval. *MCC - Matthews correlation coefficient*

Finding	Metric	Reader 1	Reader 2	Llama-2-70B
limited diagnostic quality	MCC	0.64 (0.41;0.85)	0.47 (0.17;0.75)	0.35 (0.06;0.64)
	F1	0.38 (0.22;0.53)	0.71 (0.60;0.82)	0.75 (0.65;0.84)
	Sensitivity	0.81 (0.62;0.98)	0.88 (0.73;1.00)	0.88 (0.72;1.00)
	Specificity	0.60 (0.20;0.96)	0.60 (0.21;0.96)	0.60 (0.21;0.96)
diaphragm elevation	MCC	0.71 (0.55;0.87)	0.76 (0.61;0.90)	0.80 (0.66;0.93)
	F1	0.94 (0.89;0.98)	0.94 (0.90;0.98)	0.95 (0.91;0.99)
	Sensitivity	0.92 (0.87;0.97)	0.92 (0.87;0.97)	0.93 (0.89;0.98)
	Specificity	0.86 (0.78;0.93)	0.87 (0.79;0.94)	0.88 (0.81;0.95)
diaphragm flattening	MCC	0.82 (0.72;0.91)	0.81 (0.72;0.90)	0.86 (0.78;0.94)
	F1	0.81 (0.66;0.95)	0.93 (0.83;1.00)	0.93 (0.83;1.00)
	Sensitivity	0.90 (0.78;1.00)	0.85 (0.71;0.98)	0.85 (0.71;0.98)
	Specificity	0.87 (0.79;0.94)	0.87 (0.79;0.94)	0.88 (0.81;0.95)
pneumoperitoneum	MCC	0.62 (0.33;0.89)	0.58 (0.30;0.85)	0.69 (0.41;0.95)
	F1	0.85 (0.71;0.98)	0.90 (0.77;1.00)	0.90 (0.77;1.00)
	Sensitivity	0.91 (0.86;0.96)	0.95 (0.91;0.99)	0.97 (0.94;1.00)
	Specificity	0.70 (0.47;0.91)	0.55 (0.33;0.77)	0.61 (0.41;0.81)
pneumothorax	MCC	0.70 (0.43;0.96)	0.74 (0.48;0.95)	0.74 (0.49;0.96)
	F1	0.70 (0.38;0.98)	0.53 (0.15;0.89)	0.53 (0.15;0.89)
	Sensitivity	0.84 (0.72;0.96)	0.86 (0.75;0.96)	0.84 (0.72;0.94)
	Specificity	0.79 (0.64;0.93)	0.74 (0.57;0.90)	0.70 (0.52;0.86)
pleural effusion	MCC	0.81 (0.59;0.99)	0.63 (0.34;0.91)	0.63 (0.34;0.90)
	F1	0.74 (0.49;0.96)	0.58 (0.26;0.88)	0.58 (0.26;0.88)
	Sensitivity	0.50 (0.20;0.79)	0.75 (0.55;0.94)	0.75 (0.55;0.94)
	Specificity	0.38 (-0.00;0.81)	0.38 (-0.00;0.81)	0.38 (-0.00;0.82)
atelectasis	MCC	0.74 (0.69;0.79)	0.74 (0.69;0.79)	0.75 (0.70;0.80)
	F1	0.64 (0.41;0.86)	0.44 (0.14;0.73)	0.31 (0.04;0.60)
	Sensitivity	0.33 (0.16;0.50)	0.75 (0.65;0.85)	0.78 (0.68;0.87)
	Specificity	0.81 (0.62;0.98)	0.88 (0.73;1.00)	0.88 (0.72;1.00)
lung consolidation/infiltrates	MCC	0.58 (0.16;0.95)	0.58 (0.18;0.97)	0.58 (0.18;0.96)
	F1	0.72 (0.56;0.88)	0.78 (0.64;0.91)	0.81 (0.68;0.93)
	Sensitivity	0.96 (0.94;0.99)	0.96 (0.94;0.99)	0.97 (0.94;0.99)
	Specificity	0.96 (0.93;0.98)	0.96 (0.94;0.98)	0.97 (0.94;0.99)
pulmonary edema	MCC	0.89 (0.82;0.95)	0.90 (0.83;0.95)	0.91 (0.85;0.96)
	F1	0.85 (0.77;0.93)	0.85 (0.77;0.92)	0.89 (0.82;0.95)

	Sensitivity	0.82 (0.67;0.95)	0.93 (0.84;1.00)	0.93 (0.84;1.00)
	Specificity	0.90 (0.78;1.00)	0.86 (0.71;0.98)	0.86 (0.71;0.98)
lung hyperexpansion	MCC	0.90 (0.84;0.95)	0.90 (0.84;0.95)	0.91 (0.85;0.96)
	F1	0.61 (0.30;0.90)	0.55 (0.25;0.84)	0.68 (0.38;0.95)
	Sensitivity	0.86 (0.71;0.98)	0.90 (0.78;1.00)	0.90 (0.78;1.00)
	Specificity	0.95 (0.92;0.98)	0.97 (0.95;0.99)	0.98 (0.96;1.00)
lung emphysema	MCC	0.69 (0.45;0.91)	0.56 (0.34;0.78)	0.61 (0.40;0.82)
	F1	0.70 (0.42;0.96)	0.73 (0.47;0.96)	0.73 (0.47;0.97)
	Sensitivity	0.70 (0.35;0.98)	0.52 (0.13;0.89)	0.52 (0.13;0.88)
	Specificity	0.85 (0.73;0.96)	0.87 (0.77;0.97)	0.85 (0.74;0.95)
pulmonary vascular congestion/volume overload	MCC	0.80 (0.66;0.94)	0.75 (0.58;0.91)	0.71 (0.55;0.88)
	F1	0.81 (0.57;0.99)	0.63 (0.33;0.91)	0.63 (0.33;0.90)
	Sensitivity	0.73 (0.47;0.97)	0.57 (0.24;0.87)	0.57 (0.23;0.87)
	Specificity	0.48 (0.16;0.77)	0.75 (0.53;0.95)	0.75 (0.54;0.95)
lung fibrosis	MCC	0.36 (0.00;0.80)	0.36 (0.00;0.81)	0.36 (0.00;0.81)
	F1	0.74 (0.70;0.79)	0.75 (0.70;0.79)	0.75 (0.71;0.80)
	Sensitivity	0.78 (0.54;0.98)	0.33 (0.07;0.60)	0.22 (0.01;0.46)
	Specificity	0.21 (0.08;0.34)	0.91 (0.82;0.99)	0.97 (0.92;1.00)
pulmonary nodules/mass	MCC	0.78 (0.54;0.99)	0.89 (0.70;1.00)	0.89 (0.70;1.00)
	F1	0.67 (0.25;1.00)	0.67 (0.25;1.00)	0.67 (0.24;1.00)
	Sensitivity	0.63 (0.42;0.83)	0.79 (0.62;0.95)	0.84 (0.69;0.98)
	Specificity	0.96 (0.93;1.00)	0.95 (0.91;0.99)	0.96 (0.93;1.00)
cardiomegaly	MCC	0.94 (0.90;0.98)	0.96 (0.93;0.99)	0.98 (0.96;1.00)
	F1	0.81 (0.71;0.91)	0.92 (0.84;0.98)	0.94 (0.87;0.99)
	Sensitivity	0.88 (0.78;0.96)	0.93 (0.85;0.99)	0.93 (0.85;0.99)
	Specificity	0.80 (0.62;0.97)	0.93 (0.82;1.00)	0.93 (0.82;1.00)
mediastinal widening	MCC	0.91 (0.76;1.00)	0.91 (0.76;1.00)	0.91 (0.75;1.00)
	F1	0.94 (0.88;0.99)	0.92 (0.85;0.98)	0.92 (0.84;0.98)
	Sensitivity	0.80 (0.49;1.00)	0.80 (0.49;1.00)	0.80 (0.49;1.00)
	Specificity	0.91 (0.76;1.00)	0.91 (0.75;1.00)	0.91 (0.76;1.00)
mediastinal shift	MCC	0.91 (0.86;0.96)	0.96 (0.92;0.99)	0.99 (0.97;1.00)
	F1	0.60 (0.33;0.86)	0.70 (0.45;0.95)	0.80 (0.58;0.99)
	Sensitivity	0.67 (0.35;0.97)	0.83 (0.57;1.00)	0.83 (0.57;1.00)
	Specificity	0.75 (0.39;1.00)	0.50 (0.10;0.90)	0.50 (0.10;0.90)
pneumomediastinum	MCC	0.79 (0.62;0.95)	0.95 (0.85;1.00)	0.95 (0.86;1.00)
	F1	0.81 (0.64;0.97)	0.69 (0.48;0.89)	0.75 (0.56;0.94)
	Sensitivity	0.83 (0.57;1.00)	0.67 (0.34;0.97)	0.67 (0.35;0.97)
	Specificity	0.83 (0.56;1.00)	0.50 (0.16;0.84)	0.50 (0.16;0.84)

aortic calcifications	MCC	0.38 (0.09;0.67)	0.88 (0.67;1.00)	0.88 (0.67;1.00)
	F1	0.50 (0.04;0.98)	0.50 (0.00;0.94)	0.50 (0.06;1.00)
	Sensitivity	0.75 (0.70;0.80)	0.79 (0.74;0.84)	0.80 (0.75;0.85)
	Specificity	0.99 (0.98;1.00)	0.97 (0.95;0.99)	0.97 (0.95;0.99)
degenerative bone changes	MCC	0.87 (0.82;0.91)	0.98 (0.96;1.00)	0.99 (0.98;1.00)
	F1	0.99 (0.98;1.00)	0.99 (0.99;1.00)	0.99 (0.99;1.00)
	Sensitivity	1.00 (0.99;1.00)	1.00 (0.99;1.00)	1.00 (0.99;1.00)
	Specificity	0.96 (0.94;0.99)	0.98 (0.96;1.00)	0.98 (0.97;1.00)
bone fractures	MCC	0.98 (0.95;1.00)	0.97 (0.94;0.99)	0.98 (0.95;1.00)
	F1	0.94 (0.90;0.98)	0.96 (0.93;0.99)	0.98 (0.95;1.00)
	Sensitivity	0.95 (0.91;0.98)	0.97 (0.95;0.99)	0.98 (0.96;1.00)
	Specificity	0.97 (0.94;0.99)	0.98 (0.96;1.00)	0.98 (0.96;1.00)
subcutaneous emphysema	MCC	0.98 (0.97;1.00)	0.99 (0.99;1.00)	0.99 (0.99;1.00)
	F1	0.99 (0.99;1.00)	0.99 (0.99;1.00)	0.99 (0.99;1.00)
	Sensitivity	0.98 (0.96;1.00)	0.97 (0.95;0.99)	0.97 (0.95;0.99)
	Specificity	1.00 (0.99;1.00)	1.00 (0.99;1.00)	1.00 (0.99;1.00)
soft tissue edema	MCC	0.99 (0.99;1.00)	0.99 (0.99;1.00)	0.99 (0.99;1.00)
	F1	0.94 (0.89;0.97)	0.97 (0.94;0.99)	0.99 (0.98;1.00)
	Sensitivity	0.98 (0.96;1.00)	0.99 (0.97;1.00)	0.99 (0.98;1.00)
	Specificity	0.99 (0.98;1.00)	1.00 (0.99;1.00)	1.00 (0.99;1.00)
pneumothorax	MCC	1.00 (0.99;1.00)	0.99 (0.98;1.00)	0.99 (0.98;1.00)
	F1	0.98 (0.96;1.00)	0.99 (0.98;1.00)	0.99 (0.98;1.00)
	Sensitivity	0.98 (0.97;1.00)	0.98 (0.95;0.99)	0.98 (0.96;1.00)
	Specificity	1.00 (0.99;1.00)	0.99 (0.98;1.00)	0.99 (0.98;1.00)
**All top-level findings**	MCC	1.00 (0.99;1.00)	0.99 (0.97;1.00)	0.99 (0.97;1.00)
	F1	0.98 (0.96;0.99)	1.00 (0.99;1.00)	0.99 (0.99;1.00)
	Sensitivity	1.00 (0.99;1.00)	1.00 (0.99;1.00)	1.00 (0.99;1.00)
	Specificity	0.98 (0.97;0.98)	0.98 (0.98;0.99)	0.99 (0.99;0.99)

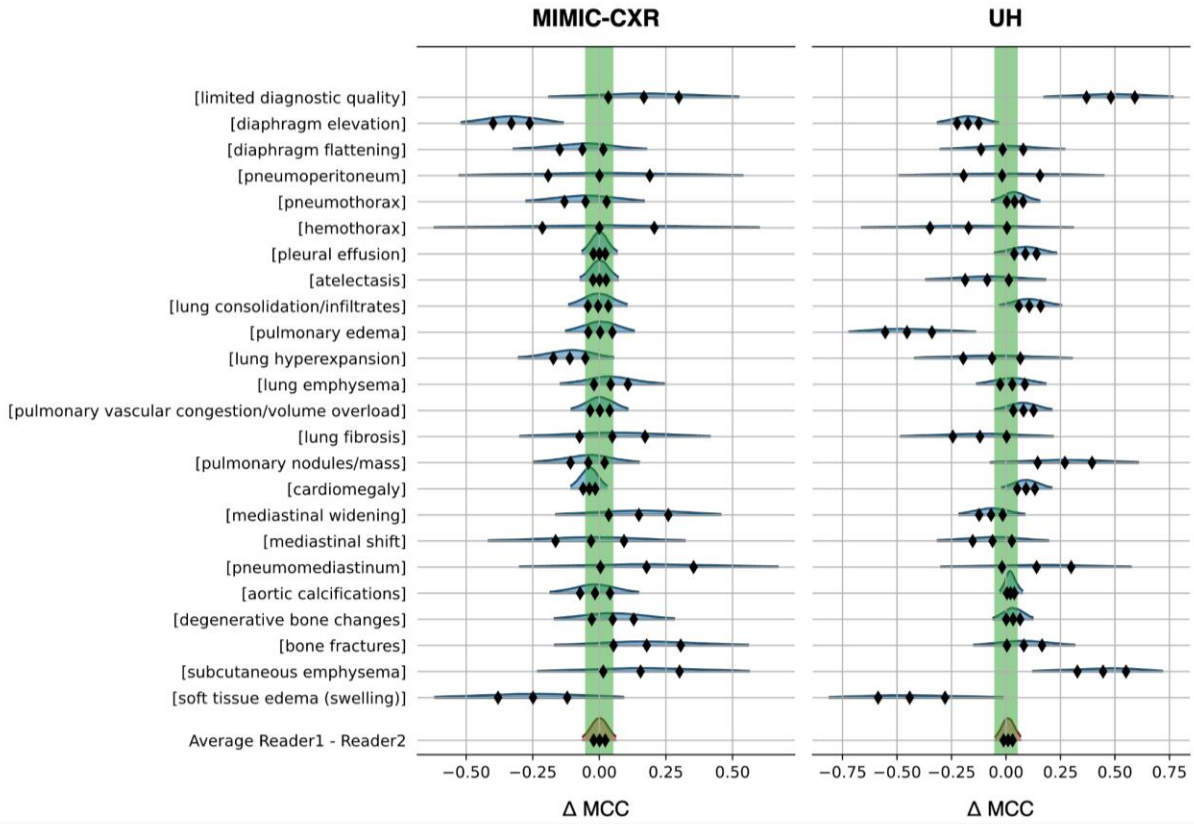

Figure S5. Detailed distribution of pairwise differences in Matthews correlation coefficient (MCC) between Reader 1 and Reader 2. The kernel density plot shows the posterior distribution of the MCC pairwise differences with the 94% highest density interval. Rhomboid markers denote quartiles. The green vertical shaded area is the region of practical equivalence (-0.05, 0.05). The red distributions represent the cumulative differences across all labels. *MIMIC-CXR - MIMIC Chest X-ray cohort, UH - University Hospital cohort*

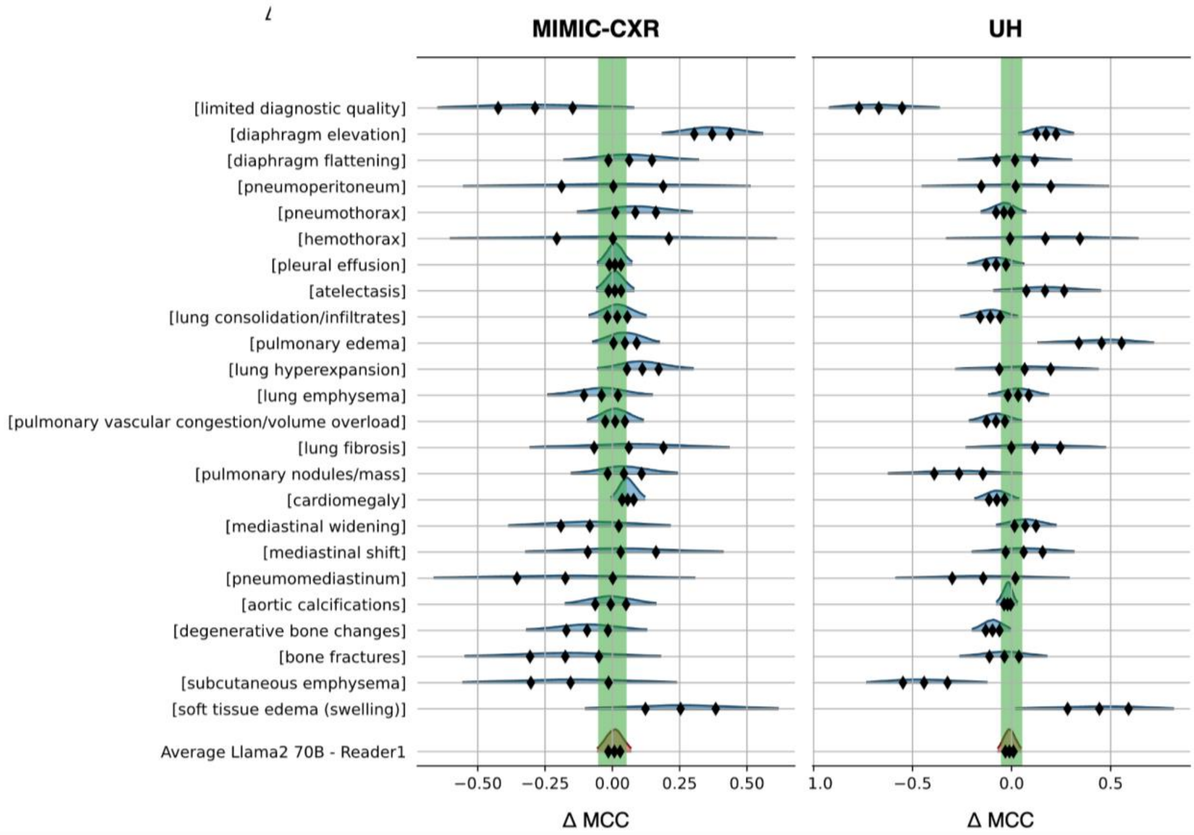

Figure S6. Detailed distribution of pairwise differences in Matthews correlation coefficient (MCC) between Llama-2-70B and Reader 1. The kernel density plot shows the posterior distribution of the MCC pairwise differences with the 94% highest density interval. Rhomboid markers denote quartiles. The green vertical shaded area is the region of practical equivalence (-0.05, 0.05). The red distributions represent the cumulative differences across all labels. *MIMIC-CXR - MIMIC Chest X-ray cohort, UH - University Hospital cohort*

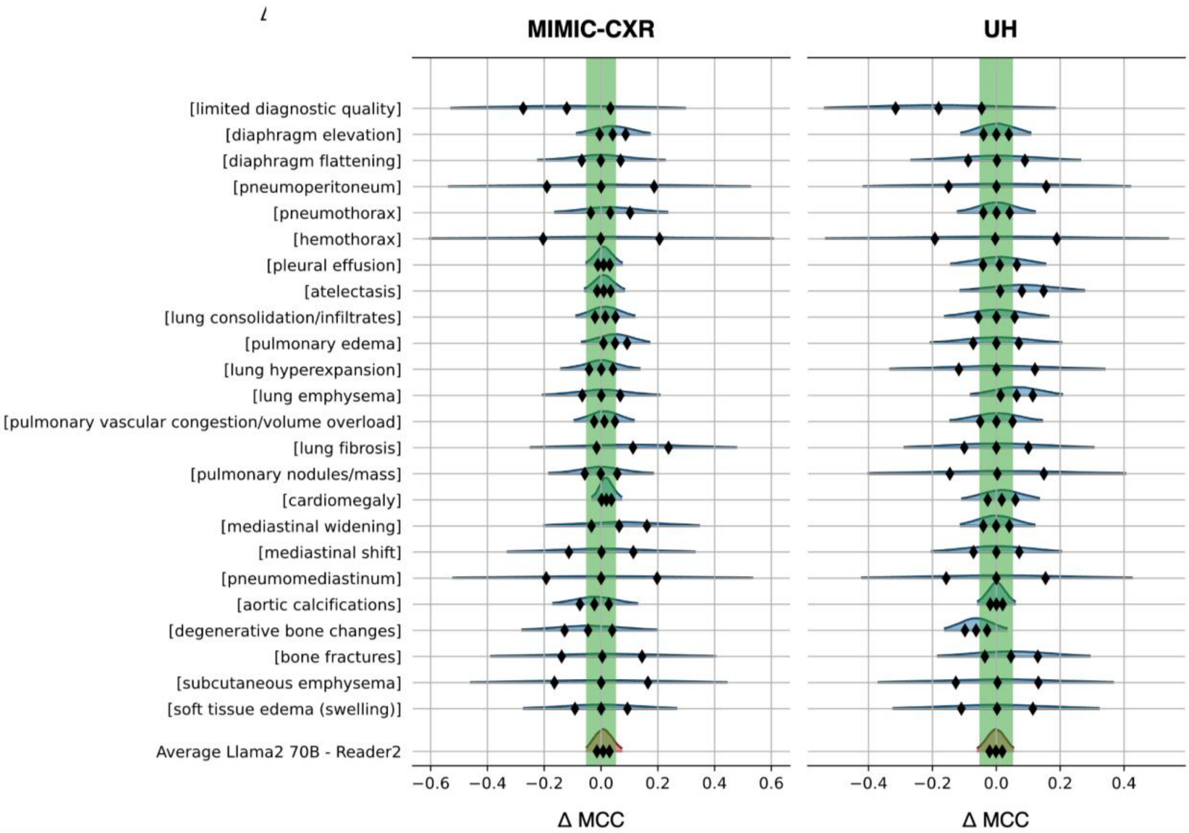

Figure S7. Detailed distribution of pairwise differences in Matthews correlation coefficient (MCC) between Llama-2-70B and Reader 2. The kernel density plot shows the posterior distribution of the MCC pairwise differences with the 94% highest density interval. Rhomboid markers denote quartiles. The green vertical shaded area is the region of practical equivalence (-0.05, 0.05). The red distributions represent the cumulative differences across all labels. *MIMIC-CXR - MIMIC Chest X-ray cohort, UH - University Hospital cohort*
